# Elevated postoperative systemic immune-inflammation index associates with acute kidney injury after cardiac surgery: a large-scale cohort study

**DOI:** 10.3389/fcvm.2024.1430776

**Published:** 2024-10-24

**Authors:** Yihao Li, Huansen Huang, Hongbin Zhou

**Affiliations:** Department of Anesthesiology, The Second Affiliated Hospital of Guangzhou Medical University, Guangzhou, China

**Keywords:** acute kidney injury, systemic immune-inflammation, cardiac surgery, mortality, MIMIC-IV

## Abstract

**Objective:**

To investigate whether postoperative systemic immune-inflammation index (SII) is associated with acute kidney injury (AKI) after cardiac surgery.

**Methods:**

We included patients undergoing cardiac surgery from the Medical Information Mart for Intensive Care—Ⅳ database to conduct a retrospective cohort study. The outcomes are AKI, severe AKI, and 30-day mortality after cardiac surgery. Analytical techniques including receiver operating characteristic (ROC) analysis, restricted cubic splines (RCS), and multivariable logistic regression were used to assess the association between SII and outcomes. Sensitivity analyses using inverse probability of treatment weighting (IPTW) and the E-value were conducted to validate the stability of the results.

**Results:**

3,799 subjects were included in this study. We used ROC to calculate an optimal cutoff value for predicting AKI after cardiac surgery, and subsequently patients were divided into two groups based on the cutoff value (Low SII: ≤ 949 × 10^9^/L; High SII: > 949 × 10^9^/L). ROC showed moderately good performance of SII for predicting AKI, while RCS also indicated a positive association between SII and AKI. The multivariate logistic analysis further affirmed the heightened risk of AKI in patients in the high SII group (OR, 5.33; 95%CI, 4.34–6.53; *P* < 0.001). Similar associations were observed between SII and severe AKI. Sensitivity and subgroup analyses indicated the robustness of the findings.

**Conclusion:**

Elevated SII was independently associated with a higher risk of AKI in adults undergoing cardiac surgery. The potential causal relationship between postoperative SII and cardiac surgery associated AKI warrants prospective research.

## 1 Introduction

Postoperative acute kidney injury (AKI), a consequential complication, impacts 5%–81% of patients following cardiac procedures (1, 2). AKI arising from cardiac surgery correlates with heightened morbidity, mortality, extended intensive care unit (ICU) stays, and escalated hospital expenses (3, 4). Patients advancing to severe AKI face an elevated 8-fold risk of 30-day mortality (5). Even as serum creatinine recovers to presurgical levels, AKI erodes renal functional reserve, fostering chronic kidney disease or failure (6). Moreover, the hospital costs for AKI patients who did not need renal replacement therapy (RRT) were up to $26,000, while they exceeded $69,000 for those needing RRT. This resulted in an annual cost of almost $1 billion for cardiac surgery-associated AKI (CSA-AKI) in the United States (7). Early identification and intervention are essential to improve prognosis, while the Society of Thoracic Surgeons took postoperative renal failure as a major metric for cardiac procedures. Though numerous predictors for postoperative AKI have been unearthed, most lack practicality and portability. Seeking a clinically accessible and uncomplicated indicator to help prevent AKI after cardiac surgery remains significant.

The pathogenesis of postoperative CSA-AKI is multifactorial, including ischemia-reperfusion injury, hypoxia, inflammatory response, and oxidative stress (6). System inflammation assumes a pivotal role in the development of AKI after surgery. Pertinent factors in cardiac surgery like aortic cross-clamp, cardiopulmonary bypass, anemia, or blood transfusion may exacerbate inflammatory response to trigger AKI (8). Numerous inflammatory biomarkers like neutrophils (9), platelets (10), and C-reactive protein (11) have been reported to associate with AKI. Nevertheless, focusing solely on a single indicator remains insufficient for clinical practice due to susceptibility to diverse confounders. The systemic immune-inflammation index (SII), derived from neutrophil, lymphocyte, and platelet counts, presents a comprehensive yet accessible metric. SII is regarded as a more reliable and representative metric compared to traditional indicators like neutrophil-to-lymphocyte ratio (NLR) and platelet-to-lymphocyte ratio (PLR) (12, 13). It effectively captures the interplay among three distinct cell lines, enhancing the predictive accuracy for cardiovascular disease and mortality. SII has been demonstrated to indicate prognosis in patients with cardiovascular disease (14), malignancy survival (15), and AKI (16). Enhanced SII levels are associated with thrombocytosis, neutrophilia, and lymphopenia, indicating a nonspecific association between inflammation and compromised adaptive immune responses (17). Prior research suggests that inflammation induces alterations in protein metabolism, while patients undergoing major non-cardiac surgery may experience diminished resilience to acute surgical stress, culminating in impaired postoperative recovery (18). As a special procedure, cardiac surgery profoundly influences inflammatory response, potentially altering SII levels and contributing to complications and mortality trends. Patients exhibiting SII deviations might experience divergent post-cardiac surgery prognoses. However, the association between SII and post-cardiac surgery AKI development was rarely discussed.

Consequently, we sought to investigate the relationship between SII and the risk of CSA-AKI. We hypothesized that postoperative SII level was related to the development of AKI after cardiac procedure.

## 2 Methods

### 2.1 Data source

All study data were sourced from a large critical care database named Medical Information Mart for Intensive Care IV (19) (MIMIC-IV version 1.0), which comprises clinical data of ICU patients from the Beth Israel Deaconess Medical Center between 2008 and 2019. The MIMIC-IV database was approved by the Institutional Review Board (IRB) of the Massachusetts Institute of Technology and Beth Israel Deaconess Medical Center (Boston, MA, USA) and granted a waiver of informed consent. All private information was not identifiable**.** The author Yihao Li has obtained the Collaborative Institutional Training Initiative (CITI) license (Record ID: 46484149), together with the permission to use the MIMIC-IV database based on pertinent rules.

### 2.2 Population section

Patients who underwent cardiac surgery were identified from all patients in the MIMIC-IV database using International Classification of Diseases (ICD) 9th and 10th Edition procedure codes. Only data from patients’ first ICU admission were used. Exclusion criteria encompassed the following: (1) age below 18; (2) unable to determine AKI status; (3) died within 48 h after surgery; (4) malignant cancers; (5) missed SII data at ICU admission.

### 2.3 Data extraction and definition

Data were extracted from MIMIC-IV on the first day of ICU admission using Structured Query Language with pgAdmin4, including age, sex, body mass index (BMI), ethnicity, admission type, surgical type, emergency operation, co-morbidities [congestive heart failure, prior myocardial infarction, peripheral vascular disease, cerebrovascular disease, chronic pulmonary disease, liver disease, chronic kidney disease (CKD), hypertension, and diabetes], severity of illness [Sequential Organ Failure Assessment (SOFA) score, simplified Acute Physiology (SAPS) Ⅱ score], medications [angiotensin-converting-enzyme inhibitors(ACEI) or angiotensin receptor blocker (ARB), and calcium channel blocker (CCB)], and laboratory data [white blood cell (WBC), hemoglobin, platelet, glucose, baseline serum creatinine, serum creatinine, blood urea nitrogen (BUN), SII]. Laboratory data were extracted at ICU admission. Intraoperative data on packed red blood cells (PRBC) and platelets transfusion were collected. Each blood product's volume of transfusion was categorized into tertiles based on equal cohort sizes. The SII index was defined as platelet count × neutrophil count/lymphocyte count (unit: G/L) upon ICU admission. We collected patients’ comorbidities information via mapping ICD-9 and ICD-10 codes to Charlson Comorbidity Index categories (20). Cardiac surgery was designated to coronary artery bypass grafting (CABG) exclusively, valve surgery exclusively, or a combination of both (CABG + valve). Some intraoperative information, such as aortic cross-clamp time and cardiopulmonary bypass time, was not available. Less than 10% of relevant variables were missing; multiple imputations were conducted to impute missing data.

### 2.4 Outcomes

The primary outcome was AKI within 7 days after surgery. Secondary outcomes included severe AKI (defined as stage 2 or 3 AKI) and 30-day mortality after cardiac surgery. AKI was defined and staged based on the Kidney Disease Improving Global Outcomes (KDIGO) definition (21) (Supplementary Table S1). We used the minimum serum creatinine collected within 7 days before admission as baseline creatinine. The first serum creatinine measured at admission was used as baseline if pre-admission creatinine was unavailable (22). The maximum AKI stage was determined based on all postoperative creatinine values. We did not use the urine output criteria of the KDIGO because previous evidence proved that they were overly sensitive and nonspecific for postcardiac surgery patients (23, 24). For analysis purposes, patients who died within 7 days after surgery without AKI were assumed not to develop AKI (25).

### 2.5 Statistical analysis

In this study, we firstly used receiver operating characteristic (ROC) analysis to assess the predictive ability of SII for outcomes, and derive the optimal cutoff value (949 × 10^9^/L) of SII for predicting AKI using the Youden index method. Subsequently, the cohort was divided into two groups based on the SII-cutoff value: low SII group (SII ≤ 949 × 10^9^/L) and the high SII group (SII > 949 × 10^9^/L) or AKI development. Continuous variables were presented as mean ± standard deviation (SD) or median (interquartile range, IQR), and intergroup comparisons were made using *t*-test or the Mann–Whitney test based on the normality of the distribution. Categorical variables were depicted as number (percentage), and between-group comparisons were undertaken through the Chi-square or Fisher's exact test, as appropriate.

The restricted cubic splines (RCS) were used to flexibly model and visualize the association between SII and AKI, severe AKI, as well as 30-day mortality, with adjustment for variables with a *P* < 0.1 in univariate logistic regression analysis. Then we utilized a two-piecewise logistic regression model with smoothing to examine the association threshold between SII and AKI. The likelihood-ratio test was used to identify inflection points. Additionally, multivariable logistic regression models were used to identify the relationships between SII and both primary and secondary outcomes, adjusting for confounders with a *P* < 0.1 in univariate analysis. Platelet and baseline serum creatinine were not included in the multivariable regression analysis due to multicollinearity. Moreover, the inverse probability of treatment weighting (IPTW) method was used to reduce potential confounding. Weights were calculated using a multivariate logistic model with the same covariates in the primary model. The weighted logistic regression model with IPTW was used to evaluate the risk for AKI within the two designated groups. We also calculated an E-value (26) to assess the potential for unmeasured confounding between SII and AKI incidence. The E-value was used to quantify the extent of an unobserved confounding factor required to nullify the observed association between SII and AKI. Furthermore, stratification analysis was undertaken to scrutinize whether the relationship between SII and AKI differed across various subgroups classified by sex, age, surgical type, pre-existing CKD, liver disease, diabetes, PRBC transfusion, platelet transfusion, and a platelet threshold of 100 K/ul; each stratification analysis adjusted for confounding factors based on *P*-value < 0.1 in univariate analysis except the stratification factor itself.

We conducted all analyses using R Statistical Software (Version 4.2.2, The R Foundation, http://www.R-project.org) and Free Statistics analysis platform (Version 1.8, Beijing, China). We utilized several packages in R including “pROC”, “compareGroups”, “foreign”, “rms”, “survminer”, “ggplot2”, “autoReg”, and “EValue”.

### 2.6 Sensitivity analysis

To estimate the potential bias resulting from the exclusions because of missing SII data, we compared the baseline characteristics between the excluded and analyzed cohorts. We assessed the balance between the stratified cohorts using standardized bias (standardized mean difference, SMD) (27).

## 3 Results

### 3.1 Baseline characteristics

**Figure 1:**
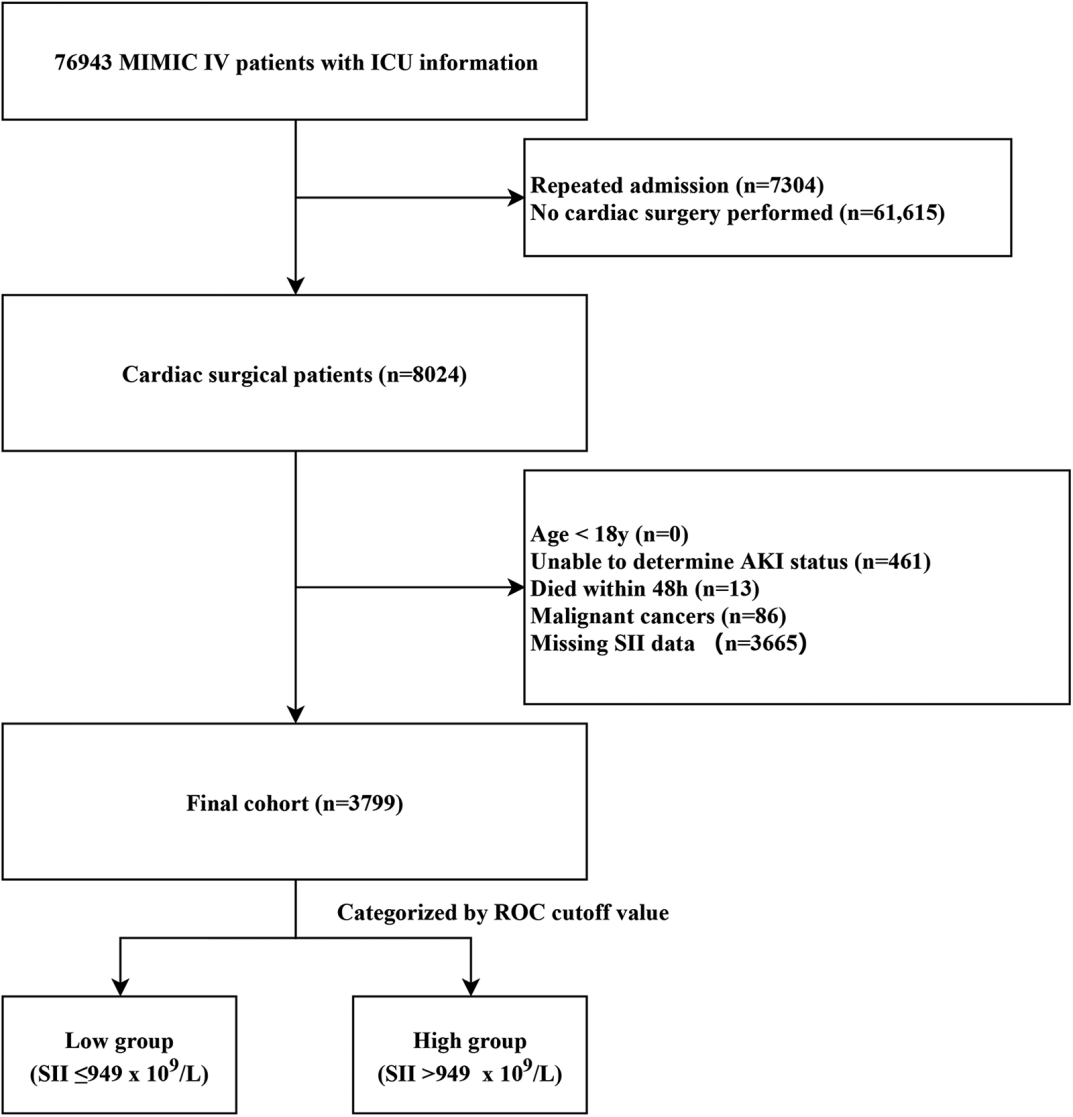
Case inclusion flowchart of the study. MIMIC, Medical Information Mart for Intensive Care; AKI, acute kidney injury; ICU, intensive care unit; ROC, receiver operator characteristic curve.

We finally enrolled 3,799 patients in this retrospective cohort study (Figure 1). We classified the final cohort into two distinct group (High SII group: SII > 949 × 10^9^/L; low SII group: SII ≤ 949 × 10^9^/L) based on the optimal SII-cutoff value for predicting CSA-AKI, and the baseline characteristics were summarized in Table 1. The mean age (IQR) of the enrolled patients was 67.4 ± 10.9 years, and 2,792 (73.5%) were men. The incidence of any-stage AKI was 21.6%, with a severe AKI incidence of 5.4%, and the 30-day mortality was 0.9%. Within the high SII group, patients were more likely to be white and admitted non-electively. High SII patients had higher BMI, more comorbidities, increased SAPS II scores, greater PRBC transfusion rates, and worse outcomes.

**Table 1:** Baseline characteristics according to SII on admission.

Variables	Total (*n* = 3,799)	SII ≤ 949 × 109/L (*n* = 2,546)	SII > 949 × 109/L (*n* = 1,253)	*P*-value
Age, years	67.4 ± 10.9	67.2 ± 10.9	67.7 ± 11.0	0.207
Sex				0.059
Female	1,007 (26.5)	699 (27.5)	308 (24.6)	
Male	2,792 (73.5)	1,847 (72.5)	945 (75.4)	
BMI, Kg/m2	29.0 ± 5.7	28.7 ± 5.4	29.7 ± 6.1	<0.001
Ethnicity				<0.001
White	2,740 (72.1)	1,788 (70.2)	952 (76)	
Black	150 (3.9)	118 (4.6)	32 (2.6)	
Hispanic	112 (2.9)	82 (3.2)	30 (2.4)	
Others	797 (21.0)	558 (21.9)	239 (19.1)	
Admission type				<0.001
Elective	508 (13.4)	375 (14.7)	133 (10.6)	
Emergency	2,292 (60.3)	1,567 (61.5)	725 (57.9)	
Urgent	999 (26.3)	604 (23.7)	395 (31.5)	
Surgical type				<0.001
CABG only	2,135 (56.2)	1,422 (55.9)	713 (56.9)	
Valve only	1,128 (29.7)	814 (32)	314 (25.1)	
Combined (CABG + Valve)	536 (14.1)	310 (12.2)	226 (18)	
Emergency operation	2,210 (58.2)	1,591 (62.5)	619 (49.4)	<0.001
Co-morbidities				
Congestive heart failure	983 (25.9)	553 (21.7)	430 (34.3)	<0.001
Prior myocardial infarction	1,147 (30.2)	679 (26.7)	468 (37.4)	<0.001
Peripheral vascular disease	496 (13.1)	322 (12.6)	174 (13.9)	0.286
Cerebrovascular disease	327 (8.6)	213 (8.4)	114 (9.1)	0.449
Chronic pulmonary disease	724 (19.1)	454 (17.8)	270 (21.5)	0.006
Liver disease	145 (3.8)	86 (3.4)	59 (4.7)	0.044
Chronic renal disease	573 (15.1)	303 (11.9)	270 (21.5)	<0.001
Hypertension	1,365 (35.9)	1,024 (40.2)	341 (27.2)	<0.001
Diabetes	1,321 (34.8)	854 (33.5)	467 (37.3)	0.023
Severity of illness				
SOFA score	3.0 (1.0, 5.0)	3.0 (1.0, 4.0)	3.0 (1.0, 5.0)	0.348
SAPS Ⅱ	36.3 ± 11.6	35.1 ± 10.8	38.7 ± 12.8	<0.001
Laboratory data				
WBC, K/ul	12.2 ± 5.1	11.2 ± 4.5	14.1 ± 5.6	<0.001
Hemoglobin, g/dl	9.9 ± 2.0	9.8 ± 1.9	10.2 ± 2.1	<0.001
Platelets, K/ul	156.9 ± 57.7	139.7 ± 45.2	191.9 ± 64.2	<0.001
Glucose, mg/dl	120.9 ± 39.6	117.8 ± 35.1	127.2 ± 46.8	<0.001
Baseline serum creatinine, mg/dl	0.9 (0.7, 1.1)	0.9 (0.7, 1.0)	0.9 (0.7, 1.1)	<0.001
BUN, mg/dl	16.0 (13.0, 20.0)	15.0 (12.0, 19.0)	17.0 (14.0, 23.0)	<0.001
Creatinine, mg/dl	0.9 (0.7, 1.1)	0.8 (0.7, 1.0)	0.9 (0.8, 1.2)	<0.001
SII, 109/L	714.6 (451.2, 1,087.5)	526.8 (369.1, 716.7)	1,265.7 (1,092.3, 1,681.3)	<0.001
Medications				
ACEI/ARB	1,013 (26.7)	593 (23.3)	420 (33.5)	<0.001
CCB	510 (13.4)	320 (12.6)	190 (15.2)	0.027
Loop diuretics	630 (16.6)	325 (12.8)	305 (24.3)	<0.001
Transfusion				
PRBC transfusion				<0.001
No transfusion	3,244 (85.4)	2,223 (87.3)	1,021 (81.5)	
≤330ml	55 (1.4)	38 (1.5)	17 (1.4)	
>330, ≤ 632 ml	251 (6.6)	160 (6.3)	91 (7.3)	
>632 ml	249 (6.6)	125 (4.9)	124 (9.9)	
Platelet transfusion				0.113
No transfusion	3,466 (91.2)	2,324 (91.3)	1,142 (91.1)	
≤256ml	111 (2.9)	75 (2.9)	36 (2.9)	
>256, ≤ 339 ml	111 (2.9)	82 (3.2)	29 (2.3)	
>339 ml	111 (2.9)	65 (2.6)	46 (3.7)	
Outcomes				
AKI	819 (21.6)	280 (11)	539 (43)	<0.001
Severe AKI	206 (5.4)	55 (2.2)	151 (12.1)	<0.001
30-day mortality	34 (0.9)	13 (0.5)	21 (1.7)	<0.001

Categorical variables are reported as No. (%), and continuous variables are reported as mean (SD) or median (interquartile range).

BMI, body mass index; CABG, coronary artery bypass grafting; SOFA, sequential organ failure Assessment; SAPS, Simplified Acute Physiology Score; WBC, white blood cell; BUN, blood urea nitrogen; ACEI, angiotensin-converting-enzyme inhibitors; ARB, angiotensin receptor blocker; CCB, calcium channel blocker; PRBC, packed red blood cells; AKI, acute kidney injury.

We also presented baseline characteristics grouped by AKI occurrence (Table 2). Most characteristics were different between non-AKI and AKI group. Patients with AKI had worse baseline conditions, with higher comorbidity prevalence, poorer SOFA and SAPS II scores, increased PRBC and platelet transfusion rates, and adverse lab parameters. However, patients without AKI had a lower proportion of non-emergency operation, hypertension, and prior myocardial infarction.

**Table 2:** Baseline characteristics of the AKI and Non-AKI groups.

Variables	Total (*n* = 3,799)	non-AKI (*n* = 2,980)	AKI (*n* = 819)	*P*-value
Age, years	67.4 ± 10.9	66.5 ± 10.8	70.7 ± 10.6	<0.001
Sex				0.05
Female	1,007 (26.5)	768 (25.8)	239 (29.2)	
Male	2,792 (73.5)	2,212 (74.2)	580 (70.8)	
BMI, Kg/m2	29.0 ± 5.7	28.9 ± 5.5	29.4 ± 6.2	0.204
Ethnicity				0.742
White	2,740 (72.1)	2,148 (72.1)	592 (72.3)	
Black	150 (3.9)	113 (3.8)	37 (4.5)	
Hispanic	112 (2.9)	87 (2.9)	25 (3.1)	
Others	797 (21.0)	632 (21.2)	165 (20.1)	
Admission type				0.002
Elective	508 (13.4)	395 (13.3)	113 (13.8)	
Emergency	2,292 (60.3)	1,839 (61.7)	453 (55.3)	
Urgent	999 (26.3)	746 (25)	253 (30.9)	
Surgical type				<0.001
CABG only	2,135 (56.2)	1,731 (58.1)	404 (49.3)	
Valve only	1,128 (29.7)	906 (30.4)	222 (27.1)	
Combined (CABG + Valve)	536 (14.1)	343 (11.5)	193 (23.6)	
Emergency operation	2,210 (58.2)	1,826 (61.3)	384 (46.9)	<0.001
Co-morbidities				
Congestive heart failure	983 (25.9)	622 (20.9)	361 (44.1)	<0.001
Prior myocardial infarction	1,147 (30.2)	852 (28.6)	295 (36)	<0.001
Peripheral vascular disease	496 (13.1)	359 (12)	137 (16.7)	<0.001
Cerebrovascular disease	327 (8.6)	232 (7.8)	95 (11.6)	<0.001
Chronic pulmonary disease	724 (19.1)	533 (17.9)	191 (23.3)	<0.001
Liver disease	145 (3.8)	98 (3.3)	47 (5.7)	0.001
Chronic renal disease	573 (15.1)	285 (9.6)	288 (35.2)	<0.001
Hypertension	1,365 (35.9)	1,182 (39.7)	183 (22.3)	<0.001
Diabetes	1,321 (34.8)	975 (32.7)	346 (42.2)	<0.001
Severity of illness				
SOFA score	3.0 (1.0, 5.0)	3.0 (1.0, 4.0)	4.0 (2.0, 6.0)	<0.001
SAPS Ⅱ	36.3 ± 11.6	34.7 ± 10.8	42.1 ± 12.7	<0.001
Laboratory data				
WBC, K/ul	12.2 ± 5.1	11.9 ± 4.8	13.1 ± 5.8	<0.001
Hemoglobin, g/dl	9.9 ± 2.0	10.0 ± 1.9	9.6 ± 2.1	<0.001
Platelets, K/ul	156.9 ± 57.7	152.0 ± 53.5	174.8 ± 68.2	<0.001
Glucose, mg/dl	120.9 ± 39.6	116.6 ± 34.6	136.5 ± 50.9	<0.001
Baseline serum creatinine, mg/dl	0.9 (0.7, 1.1)	0.9 (0.7, 1.0)	0.9 (0.7, 1.2)	<0.001
BUN, mg/dl	16.0 (13.0, 20.0)	15.0 (12.0, 19.0)	19.0 (15.0, 27.0)	<0.001
Creatinine, mg/dl	0.9 (0.7, 1.1)	0.8 (0.7, 1.0)	1.0 (0.8, 1.4)	<0.001
SII, 109/L	714.6 (451.2, 1,087.5)	630.7 (428.2, 933.0)	1,156.6 (788.4, 1,644.9)	<0.001
Medications				
ACEI/ARB	1,013 (26.7)	702 (23.6)	311 (38)	<0.001
CCB	510 (13.4)	338 (11.3)	172 (21)	<0.001
Loop diuretics	630 (16.6)	358 (12)	272 (33.2)	<0.001
Transfusion				
PRBC transfusion				<0.001
No transfusion	3,244 (85.4)	2,645 (88.8)	599 (73.1)	
≤330ml	55 (1.4)	38 (1.3)	17 (2.1)	
>330, ≤632 ml	251 (6.6)	173 (5.8)	78 (9.5)	
>632 ml	249 (6.6)	124 (4.2)	125 (15.3)	
Platelet transfusion				<0.001
No transfusion	3,466 (91.2)	2,752 (92.3)	714 (87.2)	
≤256ml	111 (2.9)	74 (2.5)	37 (4.5)	
>256, ≤339 ml	111 (2.9)	88 (3)	23 (2.8)	
>339 ml	111 (2.9)	66 (2.2)	45 (5.5)	
Outcomes				
Severe AKI	206 (5.4)	0 (0)	206 (25.2)	<0.001
30-day mortality	34 (0.9)	11 (0.4)	23 (2.8)	<0.001

Categorical variables are reported as No. (%), and continuous variables are reported as mean (SD) or median (interquartile range).

BMI, body mass index; CABG, coronary artery bypass grafting; SOFA, sequential organ failure assessmenT; SAPS, simplified acute physiology score; WBC, white blood cell; BUN, blood urea nitrogen; ACEI, angiotensin-converting-enzyme inhibitors; ARB, angiotensin receptor blocker; CCB, calcium channel blockers; PRBC, packed red blood cells; AKI, acute kidney injury.

### 3.2 Predictive ability of SII

**Figure 2:**
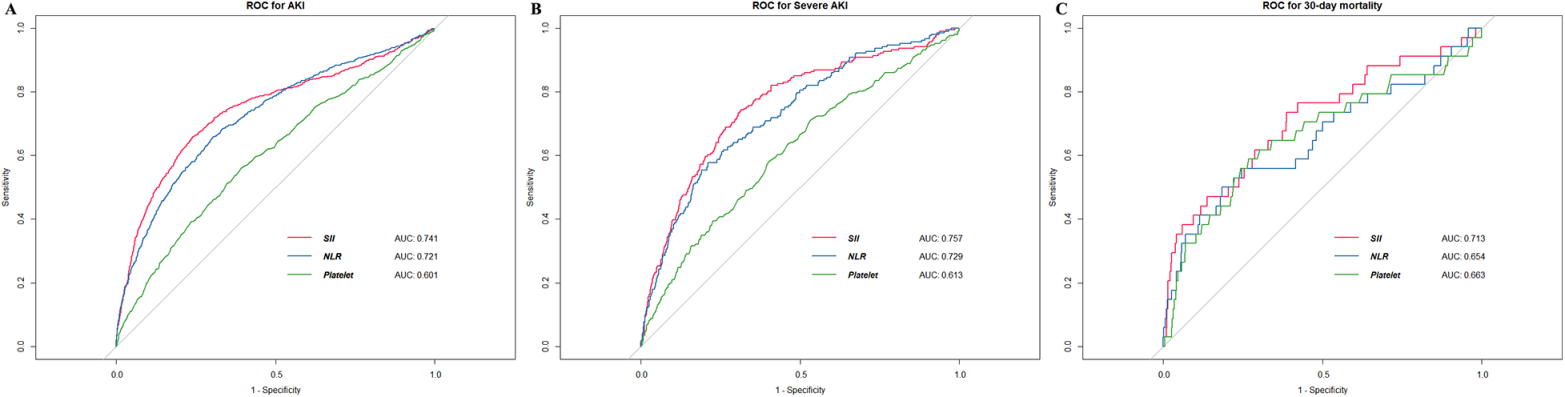
The ROC curves of SII for detecting outcomes. **(A)** AKI **(B)** severe AKI **(C)** 30-day mortality. ROC, receiver operating characteristic; AKI, acute kidney injury; AUC, area under curve; NLR, neutrophil-to-lymphocyte ratio.

We used the ROC analysis to explore the predictive value of SII, NLR, and platelet for primary and secondary outcomes. The initial SII showed moderately good predictive capacity for AKI (Figure 2A), severe AKI (Figure 2B), and 30-day mortality (Figure 2C). The cutoff value of SII for predicting AKI was 949 × 10^9^/L, with a sensitivity of 65.8% and specificity of 76.0%. Notably, SII exhibited superior area under the curve (AUC) values compared to the other indices for all observed outcomes.

### 3.3 Primary outcome

**Figure 3:**
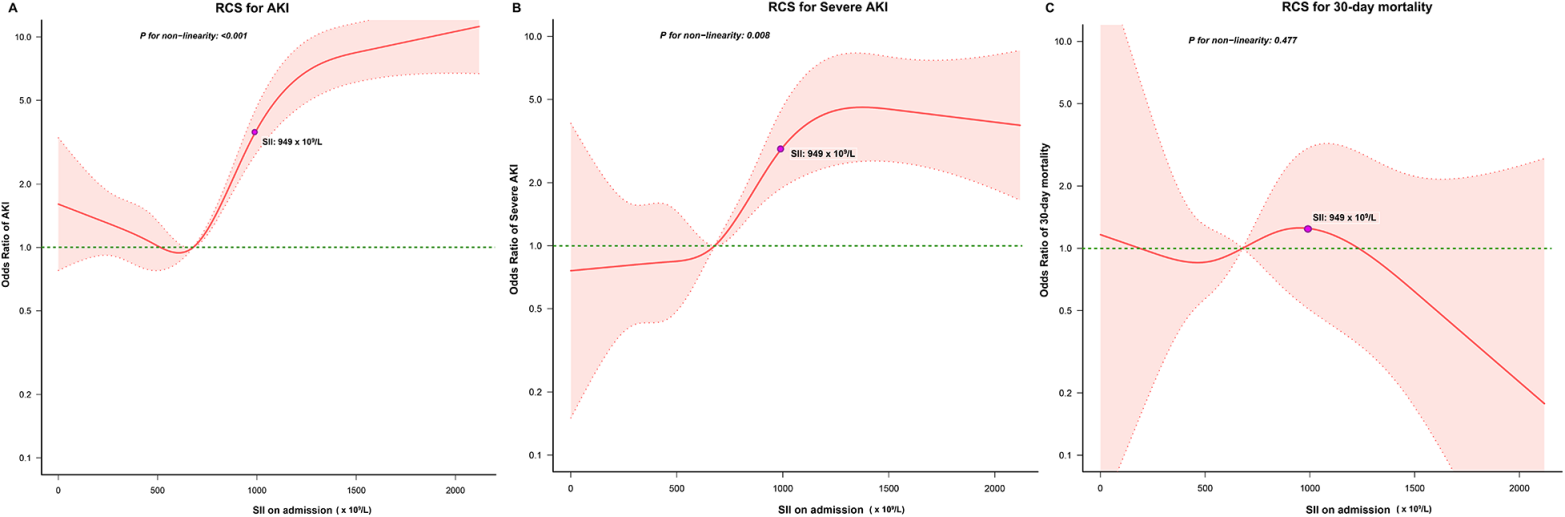
Relationship between the SII and the risk of **(A)** AKI, **(B)** severe AKI, and **(C)** 30-day mortality. The shaded area represents 95% CI. RCS, restricted cubic splines; AKI, acute kidney injury.

Our analysis revealed notable disparities in the incidence of postoperative cardiac surgery-associated AKI between patients within the high SII group and those in the low SII group (43.0% vs. 11.0%, *P* < 0.001). Subsequently, we employed RCS to model and visualize the relationship between SII and postoperative AKI (Figure 3A). When considering the initial SII as a continuous variable, we observed a “J-shape” relationship between SII and AKI within 7 days after cardiac surgery, after adjusting for covariates with a *P* < 0.1 in univariate logistic regression analysis (Supplementary Table S2). A threshold analysis was also conducted and we found an inflection point at about 563 × 10^9^/L (Table 3). On the left side of the inflection point, the relationship between SII and AKI was not statistically significant (OR, 0.999; 95%CI, 0.997∼1; *P* = 0.0976). On the right side of the inflection point, the OR was 1.002 (95%CI, 1.001∼1.002; *P* < 0.001).

**Table 3:** The non-linearity relationship between SII and CSA-AKI.

Threshold of SII	OR	95%CI	*P*-value*
≤563 × 10^9^/L	0.999	0.997∼1	0.0976
>563 × 10^9^/L	1.002	1.001∼1.002	<0.001
Non-linear test			<0.001

* Adjusted for confounding factors with *P*-value < 0.1 in univariate analysis (Supplementary Table S2).

**Figure 4:**
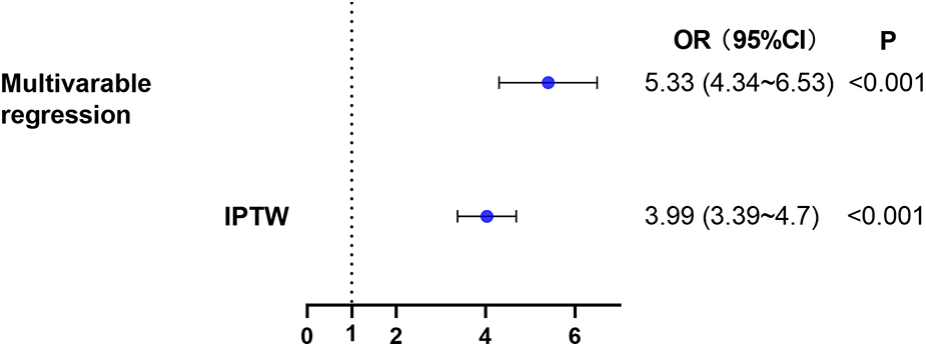
The relationship between high SII level on admission and AKI within 7 days after cardiac surgery. Two different methods were used to explore the relationship. (1) multivariable logistic regression. (2) IPTW. AKI, acute kidney injury; IPTW, inverse probability of treatment weighting.

We further examined this finding using multivariate logistic regression analyses (Figure 4 & Supplementary Table S2). Taking low SII as the reference, high SII was associated with a significant increase in risk of AKI after cardiac surgery (adjusted OR, 5.33; 95%CI, 4.34–6.53; *P* < 0.001).

Sensitivity analyses using logistic regression analysis with IPTW were consistent with the results of the main analysis (adjusted OR, 3.99; 95%CI, 3.39–4.70; *P* < 0.001). Additionally, we computed an E-value for unmeasured confounders. We found that the observed OR of 5.33 (high SII group vs. low SII group, Figure 4) for AKI incidence could be nullified by an unmeasured confounder with an OR of at least 4.05. This indicated that a considerable unmeasured confounder would be needed to negate the observed association.

**Figure 5:**
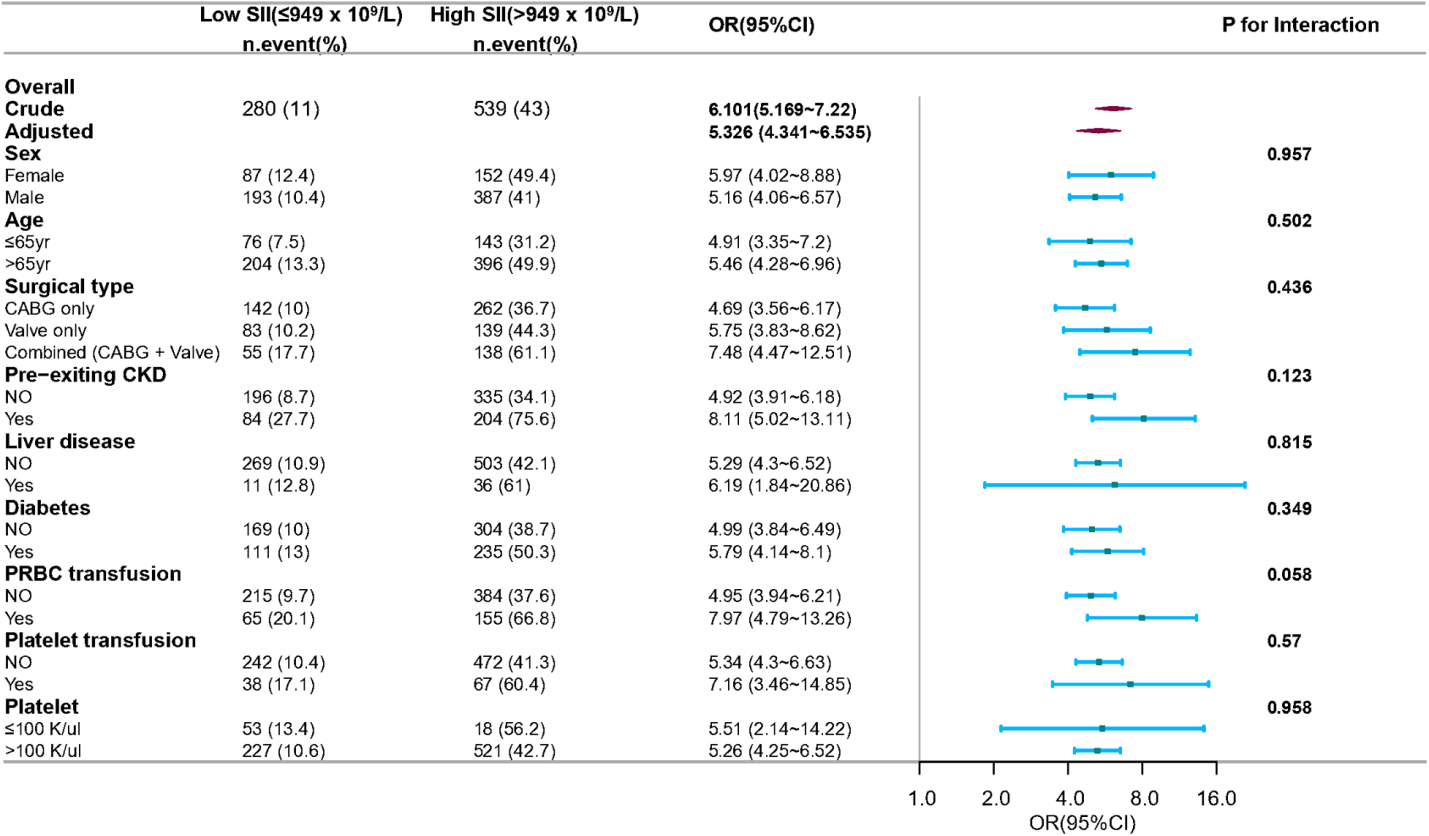
Subgroup and interaction analyses for sex, age, surgical type, pre-existing CKD, and diabetes. Multivariable logistic regression model was used to assess the influence of high SII on AKI occurrence. Each stratification analysis adjusted for confounding factors based on *P*-value < 0.1 in univariate analysis except the stratification factor itself. CABG, coronary artery bypass grafting; CKD, chronic kidney disease; PRBC, packed red blood cells.

The subgroup analysis was also conducted to ascertain the consistency of the association between SII and CSA-AKI (Figure 5). Though subgroup analysis was performed according to confounders including sex, age, surgical type, pre-existing CKD, liver disease, and diabetes, no significant interactions were detected for any subgroups (*P* for interaction > 0.05 for all). This suggested that the association between SII and postoperative AKI remained consistent across various subgroup classifications.

### 3.4 Secondary outcome

Patients in the high SII group had higher rates of postoperative severe AKI (12.1% vs. 2.2%, *P* < 0.001) and 30-day mortality (1.7% vs. 0.5%, *P* < 0.001) in comparison to those in the low SII group. We also used RCS to model and visualize the association between SII and severe AKI (Figure 3B) and 30-day mortality (Figure 3C). For severe AKI, a J-shaped curve was observed, showing a positive relationship between SII and severe AKI within 7 days after cardiac surgery, after adjusting for covariates with a *P* < 0.1in univariate logistic regression analysis (Supplementary Table S3). No significant relationship between SII and 30-day mortality was found after adjusting for covariates with a *P* < 0.1in univariate logistic regression analysis (Supplementary Table S4).

**Figure 6:**
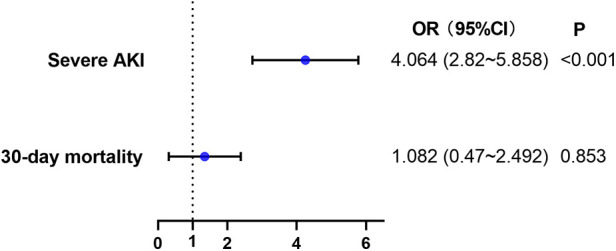
Multivariable logistic regression model assessing the association between high SII level and secondary outcomes. AKI, acute kidney injury.

Multivariate logistic regression analyses were used to examine these findings (Figure 6 & Supplementary Tables S3 & S4). High SII independently increased the incidence of severe AKI (adjusted OR, 4.064; 95%CI, 2.820–5.858; *P* < 0.001). However, there was no significant association between SII and 30-day mortality (*P* = 0.853, Figure 6 & Supplementary Table S4).

### 3.5 Sensitivity analysis

To evaluate potential selection bias stemming from exclusions because of missing SII data, we compared baseline characteristics between the excluded and analyzed cohorts (Supplementary Table S5). Patients unable to determine AKI, those who died within 48 h, and individuals with malignant cancer were excluded from the analysis. The SMD values for most variables were small, indicating minimal differences between cohorts.

## 4 Discussion

In this large-scale cohort analysis, we showed that high postoperative SII was associated with an increased risk of AKI after cardiac surgery. This correlation was similarly observed for the secondary outcome of severe AKI. Using RCS analyses, we found that SII was associated with a J-shaped incidence of both AKI and severe AKI. SII also showed moderately good predictive capacity for both AKI and severe AKI. However, high SII was not significantly associated with 30-day mortality after cardiac surgery after adjusting for potential confounders.

CSA-AKI is the most common complication after cardiac surgery. Over 2 million cardiac surgeries are performed worldwide each year and the incidence of CSA-AKI spans from 5 to 42% (3). As AKI progresses, especially when renal replacement therapy is needed, it is linked with higher mortality rates. The occurrence rate of dialysis-requiring AKI (D-AKI) after cardiac surgery ranges from 1% to 6% (28). Based on a large-scale study (29), D-AKI after cardiac surgery was linked to higher long-term mortality after discharge and increased risks of needing dialysis and developing major adverse cardiovascular events (MACE); moreover, even in patients who recovered from the need for dialysis, these outcomes were consistent. CSA-AKI is a recognized and stronger risk factor for mortality in patients who underwent cardiac procedure. Even a minor increment in serum creatinine levels after cardiac surgery signifies escalated morbidity and mortality risks, with prolonged ICU stay and augmented hospital expenditures (30). Timely identification of CSA-AKI holds paramount importance in enhancing prognostic outcomes.

The mechanisms of CSA-AKI are complex and involve multiple injury pathways, of which, aggravated inflammation may have an essential effect on the process (7). It has been reported that elevated levels of inflammatory mediators are related to the development of AKI. The platelet count stands as a potential marker capable of reflecting inflammation, with elevated counts serving as indicators of more pronounced inflammation (31). Elevated platelet count was shown to correlate with a higher risk of AKI (31). Similarly, the NLR, a gauge of inflammation, has been demonstrated to associate with AKI progression (32). Nonetheless, the sole reliance on a single index often proves inadequate since these markers are always influenced by many confounders. In this context, the SII emerges as a novel yet comprehensive index. By integrating three distinct indicators, SII potentially captures the equilibrium between inflammation, thrombosis, and immunity. Earlier investigations have demonstrated that SII had a superior ability to mirror inflammation and predict unfavorable outcomes across diverse populations, including AKI (31), cardiovascular disease (12), and cancer (33). When compared with other single indexes such as NLR and platelet, SII showed the strongest association with AKI incidence in patients undergoing coronary angiography (31). However, the association between SII levels and prognosis in patients undergoing cardiac surgery remains elusive. This study aimed to explore whether SII helped predict CSA-AKI.

The findings in this study demonstrated that the incidence of CSA-AKI was high, and elevated SII was an important risk factor for both AKI and severe AKI after cardiac procedures. A strong inflammatory response is often observed after cardiac surgery. Surgical injury, cardiopulmonary bypass, blood infusion, and ischemia-reperfusion are correlated with the activation of many inflammatory response pathways (1). Previous investigations have similarly highlighted a surge in postoperative inflammatory cytokine concentrations, correlating with subsequent AKI diagnosis and amplified mortality risk (34). The findings from this study accentuated that postoperatively elevated SII served as an indicator of exacerbated inflammation in individuals undergoing cardiac surgery. This, in turn, signified a heightened vulnerability to AKI development. Importantly, we found that SII had a better predictive performance for AKI than other single indexes, such as NLR and platelet. Moreover, our research underscored that SII was independently associated with the development of both AKI and severe AKI after cardiac surgery, even using several methods to adjust potential confounders. Given the implications of these findings, there is a need to continue to monitor SII and explore the relationship between SII and CSA-AKI further.

SII is determined using the counts of neutrophils, lymphocytes and platelets in its calculation. A decrease in platelet or neutrophil count results in a lower SII value, while critically ill patients often experience neutropenia or thrombocytopenia, which predict poor outcomes. In Figure 3A, a J-shaped relationship between SII and AKI is depicted. Lower SII theoretically suggests that the patients may be experiencing severe inflammation or immunosuppression, which could potentially explain the J-shaped relationship we observed. However, when SII was less than 563 × 10^9^/L, the relationship between SII and AKI was not statistically significant. The association between SII and prognosis in other populations has also been controversial in prior research. Jiang et al. (35) discovered a J-shaped relationship between SII and mortality in septic patients, but also found no significant difference in the increased mortality risk in the lowest quartile group. Wu et al. (36) identified a linear positive association between SII and mortality in acute ischemic stroke patients. Notably, we conducted an interaction analysis using a clinical platelet threshold of 100 K/μl and found that SII was consistently associated with AKI in both the high (>100 K/μl) and low (≤100 K/μl) platelet groups. Thus, examining the dynamic SII levels after cardiac surgery is needed to enhance the assessment of patient prognosis. Future prospective studies are needed to confirm whether there is an increased risk of AKI when SII is low.

The 30-day mortality was 0.9% among patients undergoing cardiac surgery in this research. Previous studies have consistently underscored the positive linkage between SII and mortality. Yiyuan et al. conducted a 20-year follow-up cohort study involving 42,875 US adults, revealing a close correlation between SII and both cardiovascular death and all-cause mortality (14). Lan et al. reported an association between SII and mortality in patients with AKI in a J-shaped pattern, indicative of increased risk (16). Shui et al. conducted a meta-analysis showcasing that increasing SII values were independently associated with poor survival in patients with pancreatic cancer (37). Though SII was an independent risk factor for short-term mortality across various populations, we found that SII was not associated with 30-day mortality in patients undergoing cardiac procedures after adjusting for potential confounders. This discrepancy might be attributed to varying levels of inflammation across different diseases, while there are differential degrees of correlation between inflammation and mortality across diverse populations. The insignificant association could also be influenced by the relatively low 30-day mortality rate within our study cohort. However, considering the established connection between inflammation and mortality, alongside our observations indicating SII's moderate diagnostic capacity for predicting mortality, it is advisable for subsequent investigations to closely examine the relationship between SII and mortality following cardiac surgery, as well as other pertinent outcomes.

Since previous study about SII in cardiac surgery was limited, we conducted a large-scale retrospective cohort study and found a significant association between SII and CSA-AKI. The large sample size bolstered the reliability of the findings. Nevertheless, there are deficiencies in our study. First, this retrospective observational and single-center research is subject to inherent limitations. Despite employing various methods to confirm the independent effect of SII on AKI, potential bias and confounding factors may have influenced the conclusions. Thus, we further used the E-value to assess the degree of unmeasured confounders, and we found that an unmeasured confounder was difficult to negate the observed relationship between SII and CSA-AKI. It is also hard to define an absolute range of SII to differentiate AKI outcome due to the retrospective nature. Second, we only included the initial SII value following cardiac surgery for analysis. Preoperative SII may captures the presurgical equilibrium between inflammation, thrombosis and immunity, which can be disrupted by the surgery. Regrettably, the extensive amount of missing data prevented us from incorporating preoperative SII into our research. Delta SII (postoperative SII—preoperative SII) may serve as an indicator of alterations in inflammation, thrombosis, and immunity levels, warranting further investigation in the subsequent phase of our study. Third, certain pertinent information, particularly intraoperative details like cardiopulmonary bypass time, were not available in the MIMIC database. Postoperative cardiac function is known to be associated with prognosis in patients who undergo cardiac surgery, and yet postoperative echocardiographic data were lacking in this study. The independent effect of SII on AKI, assessed without this information, may weaken its potential clinical utility. Fourth, a substantial number of patients were excluded because of missing SII data in the database, which introduced a degree of selection bias. Hence, we conducted a comparison of baseline characteristics between the excluded and analyzed cohorts and observed minimal disparity between the two groups. This suggested that missing data may have little impact on the conclusion. Fifth, platelet transfusion elevates SII directly, creating a misleading picture of inflammation and immune status as reflected by SII; hence, its utility as a marker in this context warrants further exploration. Sixth, though SII exhibited satisfactory predictive ability, the SII alone was insufficient to predict CSA-AKI. However, since we found a significant association between elevated SII and a higher risk of AKI, we suggest considering SII as a predictor when SII exceeds 563 × 10^9^/L and clinicians are advised to be highly vigilant of a higher risk of CSA-AKI when SII exceeds 949 × 10^9^/L. The causal relationship between postoperative SII and CSA-AKI warrants prospective research.

In summary, this study suggested that postoperative increased SII levels might be related to postoperative AKI in patients undergoing cardiac surgery.

## Data Availability

Publicly available datasets were analyzed in this study. This data can be found here: https://mimic.mit.edu/.

## References

[1] Wang Y Bellomo R. Cardiac surgery-associated acute kidney injury: risk factors, pathophysiology and treatment. Nat Rev Nephrol. (2017) 13:697–711. 10.1038/nrneph.2017.119 28869251

[2] Priyanka P Zarbock A Izawa J Gleason TG Renfurm RW Kellum JA. The impact of acute kidney injury by serum creatinine or urine output criteria on major adverse kidney events in cardiac surgery patients. J Thorac Cardiovasc Surg. (2021) 162:143–151.e7. 10.1016/j.jtcvs.2019.11.137 32033818

[3] Hobson CE Yavas S Segal MS Schold JD Tribble CG Layon AJ Acute kidney injury is associated with increased long-term mortality after cardiothoracic surgery. Circulation. (2009) 119:2444–53. 10.1161/CIRCULATIONAHA.108.800011 19398670

[4] Mehaffey JH Hawkins RB Byler M Charles EJ Fonner C Kron I Cost of individual complications following coronary artery bypass grafting. J Thorac Cardiovasc Surg. (2018) 155:875–882.e1. 10.1016/j.jtcvs.2017.08.144 29248284

[5] Chertow GM Levy EM Hammermeister KE Grover F Daley J. Independent association between acute renal failure and mortality following cardiac surgery. Am J Med. (1998) 104:343–8. 10.1016/S0002-9343(98)00058-8 9576407

[6] Kellum JA Lameire N. Diagnosis, evaluation, and management of acute kidney injury: a KDIGO summary (part 1). Crit Care. (2013) 17:204. 10.1186/cc11454 23394211 PMC4057151

[7] Ortega-Loubon C Tamayo E Jorge-Monjas P. Cardiac surgery-associated acute kidney injury: current updates and perspectives. J Clin Med. (2022) 11:3054. 10.3390/jcm11113054 35683442 PMC9180953

[8] Bellomo R Kellum JA Ronco C. Acute kidney injury. Lancet. (2012) 380:756–66. 10.1016/S0140-6736(11)61454-2 22617274

[9] Tadagavadi R Reeves WB. Neutrophils in cisplatin AKI-mediator or marker? Kidney Int. (2017) 92:11–3. 10.1016/j.kint.2017.03.023 28646989

[10] Jansen M Florquin S Roelofs J. The role of platelets in acute kidney injury. Nat Rev Nephrol. (2018) 14:457–71. 10.1038/s41581-018-0015-5 29760447

[11] Han SS Kim DK Kim S Chin HJ Chae DW Na KY. C-reactive protein predicts acute kidney injury and death after coronary artery bypass grafting. Ann Thorac Surg. (2017) 104:804–10. 10.1016/j.athoracsur.2017.01.075 28433221

[12] Yang Y-L Wu C-H Hsu P-F Chen S-C Huang S-S Chan WL Systemic immune-inflammation index (SII) predicted clinical outcome in patients with coronary artery disease. Eur J Clin Invest. (2020) 50:e13230. 10.1111/eci.13230 32291748

[13] Zhou Y-X Li W-C Xia S-H Xiang T Tang C Luo J-L Predictive value of the systemic immune inflammation Index for adverse outcomes in patients with acute ischemic stroke. Front Neurol. (2022) 13:836595. 10.3389/fneur.2022.836595 35370926 PMC8971364

[14] Xia Y Xia C Wu L Li Z Li H Zhang J. Systemic immune inflammation Index (SII), system inflammation response index (SIRI) and risk of all-cause mortality and cardiovascular mortality: a 20-year follow-up cohort study of 42,875 US adults. J Clin Med. (2023) 12(3):1128. 10.3390/jcm12031128 36769776 PMC9918056

[15] Chen JH Zhai ET Yuan YJ Wu KM Xu JB Peng JJ Systemic immune-inflammation index for predicting prognosis of colorectal cancer. World J Gastroenterol. (2017) 23:6261–72. 10.3748/wjg.v23.i34.6261 28974892 PMC5603492

[16] Jia L Li C Bi X Wei F Meng J Sun G Prognostic value of systemic immune-inflammation Index among critically ill patients with acute kidney injury: a retrospective cohort study. J Clin Med. (2022) 11(14):3978. 10.3390/jcm11143978 35887742 PMC9319546

[17] Aziz MH Sideras K Aziz NA Mauff K Haen R Roos D The systemic-immune-inflammation index independently predicts survival and recurrence in resectable pancreatic cancer and its prognostic value depends on bilirubin levels: a retrospective multicenter cohort study. Ann Surg. (2019) 270:139–46. 10.1097/SLA.0000000000002660 29334554

[18] Tew GA Ayyash R Durrand J Danjoux GR. Clinical guideline and recommendations on pre-operative exercise training in patients awaiting major non-cardiac surgery. Anaesthesia. (2018) 73:750–68. 10.1111/anae.14177 29330843

[19] Johnson A Bulgarelli L Shen L Gayles A Shammout A Horng S MIMIC-IV, a freely accessible electronic health record dataset. Sci Data. (2023) 10:1. 10.1038/s41597-022-01899-x 36596836 PMC9810617

[20] Charlson M Szatrowski TP Peterson J Gold J. Validation of a combined comorbidity index. J Clin Epidemiol. (1994) 47:1245–51. 10.1016/0895-4356(94)90129-5 7722560

[21] Birkelo BC Pannu N Siew ED. Overview of diagnostic criteria and epidemiology of acute kidney injury and acute kidney disease in the critically ill patient. Clin J Am Soc Nephrol. (2022) 17:717–35. 10.2215/CJN.14181021 35292532 PMC9269585

[22] De Rosa S Samoni S Ronco C. Creatinine-based definitions: from baseline creatinine to serum creatinine adjustment in intensive care. Crit Care. (2016) 20:69. 10.1186/s13054-016-1218-4 26983854 PMC4794949

[23] Katabi LJ Pu X Yilmaz HO Jia Y Leung S Duncan AE. Prognostic utility of KDIGO urine output criteria after cardiac surgery. J Cardiothorac Vasc Anesth. (2021) 35:2991–3000. 10.1053/j.jvca.2021.02.027 33744114

[24] Ryan CT Zeng Z Chatterjee S Wall MJ Moon MR Coselli JS Machine learning for dynamic and early prediction of acute kidney injury after cardiac surgery. J Thorac Cardiovasc Surg. (2023) 166(6):e551–64. 10.1016/j.jtcvs.2022.09.045 36347651 PMC10071138

[25] Xiong C Jia Y Wu X Zhao Y Yuan S Yan F Early postoperative Acetaminophen administration and severe acute kidney injury after cardiac surgery. Am J Kidney Dis. (2023) 81:675–683.e1. 10.1053/j.ajkd.2022.11.009 36586561

[26] Haneuse S VanderWeele TJ Arterburn D. Using the E-value to assess the potential effect of unmeasured confounding in observational studies. JAMA. (2019) 321:602–3. 10.1001/jama.2018.21554 30676631

[27] Austin PC. An Introduction to propensity score methods for reducing the effects of confounding in observational studies. Multivariate Behav Res. (2011) 46:399–424. 10.1080/00273171.2011.568786 21818162 PMC3144483

[28] Chen J-J Chang C-H Wu VC-C Chang S-H Hung K-C Chu P-H Long-term outcomes of acute kidney injury after different types of cardiac surgeries: a population-based study. J Am Heart Assoc. (2021) 10(9):e019718. 10.1161/JAHA.120.019718 33880935 PMC8200754

[29] Lee S Park S Kang MW Yoo H-W Han K Kim Y Postdischarge long-term cardiovascular outcomes of intensive care unit survivors who developed dialysis-requiring acute kidney injury after cardiac surgery. J Crit Care. (2019) 50:92–8. 10.1016/j.jcrc.2018.11.028 30502689

[30] Ortega-Loubon C Fernandez-Molina M Carrascal-Hinojal Y Fulquet-Carreras E. Cardiac surgery-associated acute kidney injury. Ann Card Anaesth. (2016) 19:687–98. 10.4103/0971-9784.191578 27716701 PMC5070330

[31] Jiang H Li D Xu T Chen Z Shan Y Zhao L Systemic immune-inflammation Index predicts contrast-induced acute kidney injury in patients undergoing coronary angiography: a cross-sectional study. Front Med (Lausanne). (2022) 9:841601. 10.3389/fmed.2022.841601 35372392 PMC8965764

[32] Wei W Huang X Yang L Li J Liu C Pu Y Neutrophil-to-Lymphocyte ratio as a prognostic marker of mortality and disease severity in septic acute kidney injury patients: a retrospective study. Int Immunopharmacol. (2023) 116:109778. 10.1016/j.intimp.2023.109778 36738677

[33] Yang R Chang Q Meng X Gao N Wang W. Prognostic value of systemic immune-inflammation index in cancer: a meta-analysis. J Cancer. (2018) 9:3295–302. 10.7150/jca.25691 30271489 PMC6160683

[34] Bruins P Te VH Yazdanbakhsh AP Jansen PG van Hardevelt FW de Beaumont EM Activation of the complement system during and after cardiopulmonary bypass surgery: postsurgery activation involves C-reactive protein and is associated with postoperative arrhythmia. Circulation. (1997) 96:3542–8. 10.1161/01.CIR.96.10.3542 9396453

[35] Jiang D Bian T Shen Y Huang Z. Association between admission systemic immune-inflammation index and mortality in critically ill patients with sepsis: a retrospective cohort study based on MIMIC-IV database. Clin Exp Med. (2023) 23:3641–50. 10.1007/s10238-023-01029-w 36930382 PMC10022570

[36] Wu S Shi X Zhou Q Duan X Zhang X Guo H. The association between systemic immune-inflammation Index and all-cause mortality in acute ischemic stroke patients: analysis from the MIMIC-IV database. Emerg Med Int. (2022) 2022:4156489. 35959219 10.1155/2022/4156489 PMC9363175

[37] Shui Y Li M Su J Chen M Gu X Guo W. Prognostic and clinicopathological significance of systemic immune-inflammation index in pancreatic cancer: a meta-analysis of 2,365 patients. Aging (Albany NY). (2021) 13(16):20585–97. 10.18632/aging.203449 34435973 PMC8436945

