# Supplementary material for: Elevated postoperative systemic immune-inflammation index associates with acute kidney injury after cardiac surgery: a large-scale cohort study
Source: Front Cardiovasc Med. 2024 Oct 24;11:1430776. doi: 10.3389/fcvm.2024.1430776 (PMC11540797; doi:10.3389/fcvm.2024.1430776)
Supplement: Supplementary file 1 [file Table1.docx]

**Table S1.** The Kidney Disease Improving Global Outcomes (KDIGO) definition of AKI (Creatinine Criteria)

| **AKI stage** | **Serum creatinine** |
| --- | --- |
| Stage 1 | 1.5-1.9x baseline or 0.3 mg/dL increase over 48 h |
| Stage 2 | 2.0-2.9x baseline |
| Stage 3 | 3.0x baseline or 4.0 mg/dL or new renal replacement therapy |

Abbreviations: AKI, acute kidney injury.

**Table S2.** Association between SII and AKI within 7 days after cardiac surgery.

|  | **Univariate analysis** | | **Multivariate analysis** | |
| --- | --- | --- | --- | --- |
| **Variable** | **OR(95%CI)** | ***P*-value** | **OR(95%CI)** | ***P*-value** |
| **SII** |  |  |  |  |
| ≤ 949 × 10^9^/L | Ref. |  | Ref. |  |
| > 949 × 10^9^/L | 6.109 (5.169~7.22) | <0.001 | 5.326 (4.341~6.535) | <0.001 |
| **Age, years** | 1.04 (1.032~1.048) | <0.001 | 1.028 (1.017~1.039) | <0.001 |
| **Sex** |  |  |  |  |
| Female | Ref. |  | Ref. |  |
| Male | 0.843 (0.71~1) | 0.05 | 0.874 (0.69~1.108) | 0.266 |
| **BMI, Kg/m2** | 1.015 (1.001~1.028) | 0.034 | 1.011 (0.993~1.028) | 0.228 |
| Ethnicity |  |  |  |  |
| White | Ref. |  |  |  |
| Black | 1.188 (0.811~1.741) | 0.3769 |  |  |
| Hispanic | 1.043 (0.662~1.642) | 0.8569 |  |  |
| Others | 0.947 (0.78~1.15) | 0.5843 |  |  |
| Admission type |  |  |  |  |
| Elective | Ref. |  |  |  |
| Emergency | 0.861 (0.682~1.087) | 0.2083 |  |  |
| Urgent | 1.185 (0.92~1.527) | 0.1876 |  |  |
| **Surgical type** |  |  |  |  |
| CABG only | Ref. |  | Ref. |  |
| Valve only | 1.05 (0.875~1.26) | 0.6009 | 1.099 (0.85~1.423) | 0.471 |
| Combined (CABG + Valve) | 2.411 (1.96~2.965) | <0.001 | 1.535 (1.173~2.01) | 0.002 |
| **Emergency operation** | 0.558 (0.477~0.652) | <0.001 | 0.754 (0.609~0.934) | 0.010 |
| **Congestive heart failure** | 2.988 (2.537~3.52) | <0.001 | 1.262 (0.992~1.605) | 0.058 |
| **Prior myocardial infarction** | 1.406 (1.194~1.656) | <0.001 | 0.896 (0.714~1.124) | 0.342 |
| **Peripheral vascular disease** | 1.467 (1.184~1.817) | <0.001 | 1.123 (0.853~1.478) | 0.410 |
| **Cerebrovascular disease** | 1.554 (1.208~2) | <0.001 | 1.198 (0.872~1.646) | 0.265 |
| **Chronic pulmonary disease** | 1.396 (1.158~1.684) | <0.001 | 1.042 (0.823~1.318) | 0.735 |
| **Liver disease** | 1.79 (1.253~2.558) | 0.001 | 1.29 (0.804~2.07) | 0.291 |
| **Chronic renal disease** | 5.129 (4.248~6.192) | <0.001 | 1.693 (1.267~2.263) | 0.000 |
| **Hypertension** | 0.438 (0.366~0.524) | <0.001 | 0.946 (0.751~1.19) | 0.633 |
| **Diabetes** | 1.504 (1.284~1.762) | <0.001 | 0.782 (0.62~0.987) | 0.039 |
| **SOFA score** | 1.235 (1.196~1.274) | <0.001 | 1.092 (1.047~1.139) | <0.001 |
| **SAPS Ⅱ** | 1.053 (1.046~1.06) | <0.001 | 1.018 (1.008~1.027) | 0.000 |
| **WBC, K/uL** | 1.045 (1.03~1.061) | <0.001 | 0.999 (0.98~1.018) | 0.905 |
| **Hemoglobin, g/dl** | 0.897 (0.861~0.934) | <0.001 | 0.911 (0.863~0.962) | 0.001 |
| **Glucose, mg/dL** | 1.011 (1.009~1.013) | <0.001 | 1.011 (1.008~1.013) | <0.001 |
| **Creatinine, mg/dl** | 4.152 (3.363~5.125) | <0.001 | 1.587 (1.248~2.019) | 0.000 |
| **BUN, mg/dl** | 1.082 (1.072~1.092) | <0.001 | 1.011 (0.996~1.026) | 0.154 |
| **ACEI/ ARB** | 1.987 (1.685~2.342) | <0.001 | 1.101 (0.88~1.378) | 0.398 |
| **CCB** | 2.078 (1.697~2.545) | <0.001 | 1.192 (0.905~1.569) | 0.211 |
| **Loop diuretics** | 3.642 (3.034~4.372) | <0.001 | 1.224 (0.93~1.612) | 0.149 |
| **PRBC transfusion** |  |  |  |  |
| No transfusion | Ref. |  | Ref. |  |
| ≤330ml | 1.975 (1.107~3.524) | 0.0211 | 1.059 (0.513~2.189) | 0.877 |
| >330, ≤632 ml | 1.991 (1.502~2.639) | <0.001 | 1.101 (0.768~1.578) | 0.601 |
| >632 ml | 4.451 (3.419~5.795) | <0.001 | 1.545 (1.059~2.256) | 0.024 |
| **Platelet transfusion** |  |  |  |  |
| No transfusion | Ref. |  | Ref. |  |
| ≤256ml | 1.927 (1.288~2.884) | 0.0014 | 1.794 (1.087~2.96) | 0.022 |
| >256, ≤339 ml | 1.007 (0.632~1.606) | 0.9753 | 0.65 (0.352~1.202) | 0.170 |
| >339 ml | 2.628 (1.783~3.873) | <0.001 | 1.768 (1.041~3.002) | 0.035 |

**Note:** Covariates with univariate *p*-value <0.1 were shown in bold and included into multivariate analysis.

**Abbreviations:** BMI, body mass index; CABG, coronary artery bypass grafting; SOFA, Sequential Organ Failure Assessment; SAPS, Simplified Acute Physiology Score; WBC, white blood cell; BUN, blood urea nitrogen; ACEI, angiotensin-converting-enzyme inhibitors; ARB, angiotensin receptor blocker; CCB, calcium channel blocker; PRBC, packed red blood cells; AKI, acute kidney injury.

**Table S3.** Association between SII and severe AKI after cardiac surgery

|  | **Univariate analysis** | | **Multivariate analysis** | |
| --- | --- | --- | --- | --- |
| **Variable** | **OR(95%CI)** | ***P*-value** | **OR(95%CI)** | ***P*-value** |
| **SII** |  |  |  |  |
| ≤ 949 × 10^9^/L | Ref. |  | Ref. |  |
| > 949 × 10^9^/L | 6.206 (4.521~8.518) | <0.001 | 4.064 (2.82~5.858) | <0.001 |
| **Age, years** | 1.037 (1.022~1.052) | <0.001 | 1.023 (1.005~1.041) | 0.010 |
| **Sex** |  |  |  |  |
| Female | Ref. |  | Ref. |  |
| Male | 0.655 (0.487~0.88) | 0.005 | 0.717 (0.497~1.034) | 0.075 |
| **BMI, Kg/m2** | 1.024 (1~1.049) | 0.047 | 1.031 (1.002~1.06) | 0.033 |
| Ethnicity |  |  |  |  |
| White | Ref. |  |  |  |
| Black | 1.188 (0.811~1.741) | 0.377 |  |  |
| Hispanic | 1.043 (0.662~1.642) | 0.857 |  |  |
| Others | 0.947 (0.78~1.15) | 0.584 |  |  |
| Admission type |  |  |  |  |
| Elective | Ref. |  |  |  |
| Emergency | 0.861 (0.682~1.087) | 0.208 |  |  |
| Urgent | 1.185 (0.92~1.527) | 0.188 |  |  |
| **Surgical type** |  |  |  |  |
| CABG only | Ref. |  | Ref. |  |
| Valve only | 1.193 (0.857~1.661) | 0.295 | 0.934 (0.597~1.461) | 0.764 |
| Combined (CABG + Valve) | 2.185 (1.531~3.119) | <0.001 | 0.916 (0.586~1.431) | 0.699 |
| **Emergency operation** | 0.475 (0.357~0.632) | <0.001 | 0.67 (0.469~0.958) | 0.028 |
| **Congestive heart failure** | 4.052 (3.045~5.393) | <0.001 | 1.661 (1.137~2.427) | 0.009 |
| **Prior myocardial infarction** | 1.471 (1.101~1.965) | 0.009 | 0.86 (0.588~1.258) | 0.437 |
| **Peripheral vascular disease** | 2.008 (1.426~2.828) | <0.001 | 1.443 (0.954~2.184) | 0.083 |
| **Cerebrovascular disease** | 1.812 (1.202~2.73) | 0.005 | 1.311 (0.809~2.127) | 0.272 |
| **Chronic pulmonary disease** | 1.722 (1.257~2.36) | <0.001 | 1.243 (0.859~1.8) | 0.249 |
| **Liver disease** | 2.613 (1.561~4.373) | <0.001 | 1.443 (0.748~2.782) | 0.274 |
| **Chronic renal disease** | 5.107 (3.817~6.834) | <0.001 | 1.415 (0.917~2.182) | 0.117 |
| **Hypertension** | 0.265 (0.177~0.398) | <0.001 | 0.659 (0.41~1.057) | 0.084 |
| **Diabetes** | 1.427 (1.074~1.897) | 0.014 | 0.865 (0.589~1.271) | 0.459 |
| **SOFA score** | 1.383 (1.315~1.454) | <0.001 | 1.182 (1.113~1.255) | <0.001 |
| **SAPS Ⅱ** | 1.064 (1.053~1.074) | <0.001 | 1.025 (1.011~1.04) | 0.001 |
| **WBC, K/uL** | 1.057 (1.031~1.083) | <0.001 | 1.004 (0.974~1.034) | 0.812 |
| **Hemoglobin, g/dl** | 0.86 (0.796~0.929) | <0.001 | 0.917 (0.839~1.003) | 0.058 |
| **Glucose, mg/dL** | 1.008 (1.005~1.01) | <0.001 | 1.005 (1.001~1.008) | 0.008 |
| **Creatinine, mg/dl** | 1.646 (1.485~1.824) | <0.001 | 1.248 (1.085~1.436) | 0.002 |
| **BUN, mg/dl** | 1.057 (1.046~1.067) | <0.001 | 0.991 (0.973~1.009) | 0.317 |
| **ACEI/ ARB** | 1.932 (1.448~2.578) | <0.001 | 1.058 (0.733~1.526) | 0.763 |
| **CCB** | 2.377 (1.713~3.298) | <0.001 | 1.37 (0.9~2.085) | 0.142 |
| **Loop diuretics** | 3.593 (2.678~4.821) | <0.001 | 0.85 (0.561~1.286) | 0.441 |
| **PRBC transfusion** |  |  |  |  |
| No transfusion | Ref. |  | Ref. |  |
| ≤330ml | 1.909 (0.68~5.364) | 0.2198 | 0.947 (0.292~3.073) | 0.928 |
| >330, ≤632 ml | 2.339 (1.459~3.749) | 4.00E-04 | 1.245 (0.724~2.14) | 0.428 |
| >632 ml | 6.426 (4.515~9.146) | <0.001 | 1.63 (0.992~2.677) | 0.054 |
| **Platelet transfusion** |  |  |  |  |
| No transfusion | Ref. |  | Ref. |  |
| ≤256ml | 2.187 (1.151~4.155) | 0.0169 | 1.666 (0.783~3.543) | 0.185 |
| >256, ≤339 ml | 1.544 (0.74~3.224) | 0.2474 | 1.017 (0.424~2.443) | 0.969 |
| >339 ml | 4.639 (2.814~7.647) | <0.001 | 2.253 (1.123~4.52) | 0.022 |

**Note:** Covariates with univariate *p*-value <0.1 were shown in bold and included into multivariate analysis.

**Abbreviations:** BMI, body mass index; CABG, coronary artery bypass grafting; SOFA, Sequential Organ Failure Assessment; SAPS, Simplified Acute Physiology Score; WBC, white blood cell; BUN, blood urea nitrogen; ACEI, angiotensin-converting-enzyme inhibitors; ARB, angiotensin receptor blocker; CCB, calcium channel blocker; PRBC, packed red blood cells; AKI, acute kidney injury.

**Table S4.** Association between SII and 30-day mortality after cardiac surgery

|  | **Univariate analysis** | | **Multivariate analysis** | |
| --- | --- | --- | --- | --- |
| **Variable** | **OR(95%CI)** | ***P*-value** | **OR(95%CI)** | ***P*-value** |
| **SII** |  |  |  |  |
| ≤ 949 × 10^9^/L | Ref. |  | Ref. |  |
| > 949 × 10^9^/L | 3.321 (1.657~6.655) | <0.001 | 1.082 (0.47~2.492) | 0.853 |
| **Age, years** | 1.036 (1.001~1.071) | 0.043 | 1.02 (0.984~1.056) | 0.276 |
| Sex |  |  |  |  |
| Female | Ref. |  |  |  |
| Male | 0.659 (0.325~1.336) | 0.247 |  |  |
| BMI, Kg/m2 | 1.031 (0.975~1.091) | 0.283 |  |  |
| Ethnicity |  |  |  |  |
| White | 0.701 (0.094~5.198) | 0.728 |  |  |
| Black | 0 (0~Inf) | 0.98 |  |  |
| Hispanic | 0.925 (0.4~2.139) | 0.855 |  |  |
| Others | 0.701 (0.094~5.198) | 0.728 |  |  |
| Admission type |  |  |  |  |
| Elective | Ref. |  |  |  |
| Emergency | 0.997 (0.336~2.96) | 0.996 |  |  |
| Urgent | 1.532 (0.492~4.774) | 0.462 |  |  |
| Surgical type |  |  |  |  |
| CABG only | Ref. |  |  |  |
| Valve only | 1.304 (0.603~2.82) | 0.499 |  |  |
| Combined (CABG + Valve) | 1.752 (0.717~4.282) | 0.218 |  |  |
| **Emergency operation** | 0.296 (0.141~0.622) | 0.001 | 0.521 (0.223~1.215) | 0.131 |
| **Congestive heart failure** | 5.349 (2.637~10.85) | <0.001 | 1.755 (0.727~4.24) | 0.211 |
| **Prior myocardial infarction** | 2.07 (1.052~4.074) | 0.035 | 1.901 (0.879~4.108) | 0.102 |
| **Peripheral vascular disease** | 4.206 (2.093~8.456) | <0.001 | 2.394 (1.073~5.341) | 0.033 |
| **Cerebrovascular disease** | 2.791 (1.206~6.46) | 0.017 | 1.938 (0.74~5.075) | 0.178 |
| Chronic pulmonary disease | 1.78 (0.848~3.74) | 0.128 |  |  |
| **Liver disease** | 8.148 (3.623~18.327) | <0.001 | 5.873 (2.276~15.154) | <0.001 |
| Chronic renal disease | 1.465 (0.635~3.381) | 0.370 |  |  |
| **Hypertension** | 0.305 (0.118~0.789) | 0.014 | 0.866 (0.29~2.586) | 0.796 |
| Diabetes | 1.316 (0.663~2.615) | 0.432 |  |  |
| **SOFA score** | 1.376 (1.241~1.525) | <0.001 | 1.127 (0.995~1.276) | 0.061 |
| **SAPS Ⅱ** | 1.052 (1.029~1.076) | <0.001 | 1.019 (0.987~1.051) | 0.254 |
| **WBC, K/uL** | 1.058 (0.998~1.122) | 0.057 | 1.009 (0.949~1.073) | 0.774 |
| Hemoglobin, g/dl | 1.076 (0.914~1.268) | 0.380 |  |  |
| **Glucose, mg/dL** | 1.008 (1.003~1.012) | <0.001 | 1.127 (0.995~1.276) | 0.061 |
| Creatinine, mg/dl | 1.176 (0.935~1.48) | 0.166 |  |  |
| **BUN, mg/dl** | 1.039 (1.015~1.062) | 0.001 | 0.996 (0.964~1.03) | 0.818 |
| **ACEI/ ARB** | 2.78 (1.414~5.467) | 0.003 | 1.265 (0.563~2.841) | 0.569 |
| CCB | 1.386 (0.571~3.365) | 0.470 |  |  |
| **Loop diuretics** | 6.539 (3.304~12.939) | <0.001 | 2.07 (0.831~5.158) | 0.118 |
| **PRBC transfusion** |  |  |  |  |
| No transfusion | Ref. |  | Ref. |  |
| ≤330ml | 0 (0~Inf) | 0.9878 | 0 (0~Inf) | 0.985 |
| >330, ≤632 ml | 1.363 (0.316~5.887) | 0.6779 | 0.684 (0.151~3.093) | 0.622 |
| >632 ml | 9.35 (4.561~19.166) | <0.001 | 1.729 (0.631~4.734) | 0.287 |
| **Platelet transfusion** |  |  |  |  |
| No transfusion | Ref. |  | Ref. |  |
| ≤256ml | 8.127 (2.993~22.068) | <0.001 | 6.494 (2.052~20.551) | 0.002 |
| >256, ≤339 ml | 1.566 (0.208~11.777) | 0.6628 | 1.112 (0.129~9.615) | 0.923 |
| >339 ml | 13.383 (5.76~31.093) | <0.001 | 5.086 (1.548~16.709) | 0.007 |

**Note:** Covariates with univariate *p*-value <0.1 were shown in bold and included into multivariate analysis.

**Abbreviations:** BMI, body mass index; CABG, coronary artery bypass grafting; SOFA, Sequential Organ Failure Assessment; SAPS, Simplified Acute Physiology Score; WBC, white blood cell; BUN, blood urea nitrogen; ACEI, angiotensin-converting-enzyme inhibitors; ARB, angiotensin receptor blocker; CCB, calcium channel blocker; PRBC, packed red blood cells; AKI, acute kidney injury.

**Table S5.** Baseline characteristics of the cohort excluded and the cohort analyzed

| **Variables** | **Total**  **(n =7464)** | **cohort excluded**  **(n = 3665)** | **cohort analyzed**  **(n = 3799)** | **SMD** |
| --- | --- | --- | --- | --- |
| Age, years | 67.7 ± 11.4 | 68.0 ± 11.8 | 67.4 ± 10.9 | 0.054 |
| Sex |  |  |  | 0.0779 |
| Female | 2107 (28.2) | 1100 (30) | 1007 (26.5) |  |
| Male | 5357 (71.8) | 2565 (70) | 2792 (73.5) |  |
| BMI, Kg/m2 | 29.0 ± 5.8 | 29.0 ± 5.9 | 29.0 ± 5.7 | 0.0137 |
| Ethnicity |  |  |  | 0.0323 |
| White | 5430 (72.7) | 2690 (73.4) | 2740 (72.1) |  |
| Black | 291 (3.9) | 141 (3.8) | 150 (3.9) |  |
| Hispanic | 223 (3.0) | 111 (3) | 112 (2.9) |  |
| Others | 1520 (20.4) | 723 (19.7) | 797 (21) |  |
| Admission type |  |  |  | 0.081 |
| Elective | 1047 (14.0) | 539 (14.7) | 508 (13.4) |  |
| Emergency | 4357 (58.4) | 2065 (56.3) | 2292 (60.3) |  |
| Urgent | 2060 (27.6) | 1061 (28.9) | 999 (26.3) |  |
| Surgical type |  |  |  | 0.0784 |
| CABG only | 4079 (54.6) | 1944 (53) | 2135 (56.2) |  |
| Valve only | 2239 (30.0) | 1111 (30.3) | 1128 (29.7) |  |
| Combined (CABG + Valve) | 1146 (15.4) | 610 (16.6) | 536 (14.1) |  |
| **Emergency operation** | 4165 (55.8) | 1955 (53.3) | 2210 (58.2) | 0.0974 |
| **Co-morbidities** |  |  |  |  |
| Congestive heart failure | 2073 (27.8) | 1090 (29.7) | 983 (25.9) | 0.0864 |
| Prior myocardial infarction | 2219 (29.7) | 1072 (29.2) | 1147 (30.2) | 0.0206 |
| Peripheral vascular disease | 1076 (14.4) | 580 (15.8) | 496 (13.1) | 0.0788 |
| Cerebrovascular disease | 720 (9.6) | 393 (10.7) | 327 (8.6) | 0.0716 |
| Chronic pulmonary disease | 1565 (21.0) | 841 (22.9) | 724 (19.1) | 0.0956 |
| Liver disease | 296 (4.0) | 151 (4.1) | 145 (3.8) | 0.0155 |
| Chronic renal disease | 1145 (15.3) | 572 (15.6) | 573 (15.1) | 0.0145 |
| Hypertension | 2205 (29.5) | 840 (22.9) | 1365 (35.9) | 0.2885 |
| Diabetes | 2593 (34.7) | 1272 (34.7) | 1321 (34.8) | 0.0014 |
| Severity of illness |  |  |  |  |
| SOFA score | 3.0 (1.0, 4.0) | 3.0 (1.0, 4.0) | 3.0 (1.0, 5.0) | 0.0786 |
| SAPS Ⅱ | 36.5 ± 11.8 | 36.6 ± 12.0 | 36.3 ± 11.6 | 0.0309 |
| Laboratory data |  |  |  |  |
| Baseline serum creatinine, mg/dl | 0.9 (0.7, 1.1) | 0.9 (0.7, 1.1) | 0.9 (0.7, 1.1) | 0.0452 |
| BUN, mg/dl | 16.0 (13.0, 21.0) | 17.0 (13.0, 22.0) | 16.0 (13.0, 20.0) | 0.1361 |
| Creatinine, mg/dl | 0.9 (0.7, 1.1) | 0.9 (0.7, 1.1) | 0.9 (0.7, 1.1) | 0.0654 |
| **Medications** |  |  |  |  |
| ACEI/ ARB | 1841 (24.7) | 828 (22.6) | 1013 (26.7) | 0.0946 |
| CCB | 854 (11.4) | 344 (9.4) | 510 (13.4) | 0.1273 |
| Loop diuretics | 1063 (14.2) | 433 (11.8) | 630 (16.6) | 0.1369 |
| **Transfusion** |  |  |  |  |
| PRBC transfusion | 1258 (16.85) | 703 (19.18) | 555 (14.61) | 0.1223 |
| Platelet transfusion | 684 (9.16) | 351 (9.58) | 333 (8.77) | 0.0281 |
| **Outcomes** |  |  |  |  |
| AKI | 1834 (24.6) | 1015 (27.7) | 819 (21.6) | 0.1428 |
| Severe AKI | 556 (7.4) | 350 (9.5) | 206 (5.4) | 0.1573 |
| 30-day mortality | 89 (1.2) | 55 (1.5) | 34 (0.9) | 0.0557 |

**Note:** Categorical variables are reported as No. (%), and continuous variables are reported as mean (SD) or median (interquartile range).

**Abbreviations:** SMD, standard mean difference; BMI, body mass index; CABG, coronary artery bypass grafting; SOFA, Sequential Organ Failure Assessment; SAPS, Simplified Acute Physiology Score; WBC, white blood cell; BUN, blood urea nitrogen; ACEI, angiotensin-converting-enzyme inhibitors; ARB, angiotensin receptor blocker; CCB, calcium channel blocker; PRBC, packed red blood cells; AKI, acute kidney injury.
